# Influence of kyphosis in ankylosing spondylitis on cardiopulmonary functions

**DOI:** 10.1097/MD.0000000000035592

**Published:** 2023-10-27

**Authors:** Yunfei Yang, Lijun Huang, Guofeng Zhao, Jiyuan Xia, Xinqi Tian, Chang Liu, Qingfu Xia

**Affiliations:** a Beijing Da wang Lu Emergency Hospital, Spinal Surgery, Beijing, China; b Beijing Youlian Hospital, Spinal Surgery, Beijing, China; c Hebei Medical University, Shijiazhuang, China.

**Keywords:** ankylosing spondylitis (AS), cardiac structure, echocardiography, global kyphosis, pulmonary function

## Abstract

This paper aims at analyzing the characteristics of cardiopulmonary functions in the patients with ankylosing spondylitis (AS), and exploring the influence of global kyphosis (GK) on cardiopulmonary functions. Clinical data of 46 patients with AS and kyphosis, who had been admitted in our hospital from October 2021 to October 2022, were analyzed retrospectively. According to the to global kyphosis (GK) angle, 23 subjects were divided into Severe Group (GK > 95°), and 23 subjects were divided into in the Moderate Group (80° ≤ GK ≤ 95°). Cardiac structure and cardiopulmonary function parameters were compared between both groups, and the influences of GK Angle on other parameters were analyzed by Pearson or Spearman correlation analysis. The cardiac structure and function measurements in both groups were within the normal range. The pulmonary functions of both groups decreased to different extents. Correlation analysis showed that GK Angle was significantly negatively correlated with the left atrioventricular size (LAD, LVDD, LVSD) and diastolic function parameters (E/A, e’/a’) in the patients with AS (*P *< .05); GK Angle was negatively correlated with restrictive ventilation parameters in the patients with AS (*P *< .05). The GK Angle of the patients with AS affects the cardiac structure and diastolic function. The larger the GK Angle is, the smaller the left and right at ventricle diameters are. In addition, GK Angle also affects the left ventricular diastolic function. GK Angle is related to the degree of pulmonary function impairment, and the larger the GK Angle is, the worse the pulmonary function it will be.

## 1. Introduction

Ankylosing spondylitis (AS) is a chronic disease involving the axial skeleton of the body, and it is a kind of rheumatism characterized by chronic inflammatory changes in large joints, such as sacroiliac joint and hip joint, and spinal arthritis belongs to one of its subtypes, with a prevalence rate of about 2%.^[1]^ Its pathological changes are lesions in the attachment of the ligament, joint fibers or bone stiffness, vertebral osteoporosis, muscle stiffness and atrophy, and kyphosis.^[2]^ It leads to structural and functional damages and reduces in quality of life. In the Severe Group, kyphosis leads to muscle fatigue, activity related pain caused by continuous tension of spinal muscles while standing upright, inability to look straight ahead, intraperitoneal complications, and impaired respiratory function.^[3–6]^ Restricted chest wall movement and abnormal lung parenchyma are the main factors leading to pulmonary dysfunction.^[7]^ AS not only reduces the volume of thoracic cavity due to kyphosis, but also further reduces the volume of thoracic cavity due to rib compression of abdominal cavity caused by kyphosis of thoracolumbar spine, thus affecting pulmonary function. In addition, the inflammatory reaction of AS disease also causes ossification of thoracic joints and mandatory spondylitis, thus reducing thoracic compliance and further damaging pulmonary function.^[8]^ However, whether global kyphosis (GK) angle affects cardiac systolic and diastolic function in the patients with AS and thoracolumbar kyphosis is still uncertain. In this study, the pulmonary function, cardiac structure and functional characteristics of the patients with AS were retrospectively analyzed, and the relationship between GK Angle and the degree of pulmonary function impairment, whether the GK Angle of the patients with AS affects the cardiac structure, diastolic function, and the left ventricular diastolic function were explored after excluding the influence of thoracic malformation and gender.

## 2. Data and methods

### 2.1. Inclusion and exclusion criteria

Inclusive criteria: The patients with AS were confirmed; The patients’ main deformity is kyphosis, and the GK angle is ≥ 80^°^; The clinical data and various auxiliary examination data are complete. Exclusion criteria: The patients with primary rib and thoracic deformities; The patients with congenital heart disease; The patients with a history of cardiothoracic surgery; The patients with a history of spinal trauma or spinal surgery; The patients suffer from bronchial asthma, expansion and other lung diseases, or with a recent history of lung infection.

### 2.2. General information

Retrospective analysis was conducted on the patients with AS who had been admitted in *** Hospital from October 2021 to October 2022. In this study, 46 patients who were qualified for the above criteria were selected and included. According to different GK angles, 23 patients were divided into Severe Group (GK angle ≥ 95^°^) and 23 patients were divided into Moderate Group (80^°^ ≤ GK angle < 95^°^). The study was approved by the Medical Ethics Committee of the Hospital (Ethics No.: 2022052301), and all subjects had signed the informed consent.

### 2.3. Research methods

The general data of the patients, such as age, height, weight and arm length, were collected. All patients completed the whole spine X-ray examination in standing position, and the GK angle was measured on the full length anteroposterior and lateral X-ray films using Surgimap software. The Philip7C ultrasonic instrument was used to measure the cardiac structure and cardiopulmonary function parameters: Cardiac structure parameters: left atrial diameter (LAD), left ventricular diameter in end diameter (LVDD), left ventricular diameter in end systolic (LVSD), right atrial diameter, right ventricular diameter (RVD), aortic diameter (AO), main pulmonary artery diameter, inter ventricular septum thickness, left ventricular posterior wall thickness; Left ventricular systolic function parameters: ejection fraction (EF); Left ventricular diastolic function parameters: ratio of early diastolic maximum peak velocity (E peak) to late diastolic maximum peak velocity (A peak)(E/A), ratio of early diastolic velocity (e’) to late diastolic velocity (a’)(e’/a’), ratio of early diastolic maximum peak velocity (E peak) to early diastolic velocity (e’) of mitral septal annulus (E/e’). The automatic pulmonary function tester was used to measure pulmonary function parameters: restrictive ventilation function parameters: measured value of forced vital capacity (FVC), measured value/estimated value of FVC, measured value/estimated value of maximum vital capacity (VC _max_), measured value/estimated value of VC-_max_, measured value/estimated value of total lung capacity (TLC), measured value/estimated value of TLC; Obstructive ventilation function parameters: measured value of forced expiratory volume/forced vital capacity (FEV1/FVC) in the first second, measured value/predicted value of FEV1/FVC; Severity parameters of ventilation function: measured value of forced expiratory volume in the first second (FEV1), measured value/predicted value of FEV1; Small airway function parameters: MMEF75/25 measured value, MMEF75/25 measured value/predicted value, MEF75 measured value, MEF75 measured value/predicted value, MEF50 measured value, MEF50 measured value/predicted value, MEF25 measured value, MEF25 measured value/predicted value; Ventilation function parameters: measured value of carbon monoxide dispersion (DLCO), measured value/estimated value of DLCO.

### 2.4. Statistical methods

SPSS 25.0 software was used for statistical analysis. The measurement data was represented by *x ± s*. When the data were normally distributed, independent sample *t* test was used for comparison between both groups; When the data is in non-normally distributed, Mann–Whitney *U* test was used for testing. The GK angle and age of the spine were correlated with the indexes of cardiopulmonary functions. Pearson correlation analysis was used when the data were normally distributed, and Spearman rank correlation analysis was used when the data were non-normally distributed. *P* < .05 means that the difference is statistically significant.

## 3. Results

### 3.1. General information

See Table 1 for the general information of both groups of the patients. The height, arm length, weight and body mass index of the Severe Group were significantly lower than those of Moderate Group (*P* < .05), while GK angle was significantly higher than that of Moderate Group (*P* < .05). The average age of both groups was also statistically significant (*P* < .05).

**Table 1:** Comparison of general data of both groups of the patients (*x ± s*).

Index	Severe group (n = 23)	Moderate group (n = 23)	*P* value
Age (yr)	41.57 ± 8.84	36.43 ± 7.18	.040*
Height (cm)	144.28 ± 13.54	159.55 ± 7.26	<.001*
Weight (kg)	54.43 ± 5.54	66.04 ± 12.83	<.001*
BMI (kg/m^2^)	20.73 ± 3.51	23.00 ± 3.77	.043*
Arm length (cm)	162.84 ± 8.56	169.19 ± 7.30	.011*
GK angle (°)	105.96 ± 8.20	85.83 ± 4.69	<.001*

BMI = body mass index, GK = global kyphosis.

* Indicates that the comparison between both groups is statistically significant (*P* < .05).

### 3.2. Comparison of cardiac structure and cardiopulmonary function parameters between both groups

The measured results of cardiac structure of both groups are shown in Table 2. The measured values of cardiac structure and cardiopulmonary function in all patients were within normal range. The diameter of atrioventricular cavity (LAD, LVDD, LVSD, right atrial diameter, and RVD) in the Severe Group was significantly lower than that in the Moderate Group (*P* < .05), and the parameters of left ventricular diastolic function E/A, e’/a’ in the Severe Group were significantly lower than that in the Moderate Group (*P* < .05), while E/e’ was significantly higher than that in the Moderate Group (*P* < .05). There was no significant difference in the diameter (AO, main pulmonary artery diameter), ventricular wall thickness (inter ventricular septum thickness, left ventricular posterior wall thickness) and systolic function parameters (EF) between both groups (*P* > .05).

**Table 2:** Comparison of cardiac structure and function parameters between both groups (*x ± s*).

Index	Severe group (n = 23)	Moderate group (n = 23)	*P* value
LAD (mm)	29.43 ± 2.62	31.26 ± 3.10	.04*
LVDD (mm)	42.57 ± 2.92	44.57 ± 3.41	.04*
LVSD (mm)	28.30 ± 2.69	29.96 ± 2.61	.04*
RAD (mm)	30.65 ± 3.77	33.78 ± 3.73	.01*
RVD (mm)	30.22 ± 3.67	32.52 ± 3.68	.04*
AO (mm)	19.70 ± 1.78	19.70 ± 2.07	1.00
MPA (mm)	19.87 ± 2.03	20.61 ± 2.37	.27
IVS (mm)	9.43 ± 1.14	9.74 ± 1.54	.46
LVPW (mm)	9.35 ± 0.76	9.87 ± 1.23	.10
EF (%)	61.17 ± 4.45	61.78 ± 4.34	.65
E/A	1.04 ± 0.25	1.22 ± 0.32	.04*
e’/a’	1.04 ± 0.32	1.33 ± 0.58	.04*
E/e’	9.78 ± 3.02	8.08 ± 1.66	.03*

AO = aortic diameter, EF = ejection fraction, IVS = inter ventricular septum thickness, LAD = left atrial diameter, LVDD = left ventricular diameter in end diameter, LVPW = left ventricular posterior wall thickness, LVSD = left ventricular diameter in end systolic, MPA = main pulmonary artery diameter, RAD = right atrial diameter, RVD = right ventricular diameter.

* Indicates that the comparison between both groups is statistically significant (*P* < .05).

### 3.3. Comparison of pulmonary function parameters between both groups

See Table 3 for pulmonary function parameters of both groups. The pulmonary function of both groups was damaged to different extents. The restrictive ventilatory function parameters (FVC measured value, VC_max_ measured value, TLC measured value, TLC measured value/predicted value) in the Severe Group were significantly lower than those in the Moderate Group (*P* < .05), and the measured value, FEV1 measured value/predicted value in the Severe Group were significantly lower than those in the Moderate Group (*P* < .05). The small airway function parameters (MEF75 measured value, MEF50 measured value, MEF25 measured value, and MEF25 measured value/predicted value) and ventilation function indexes (DLCO measured value and DLCO measured value/predicted value) in the Severe Group were significantly lower than those in the Moderate Group (*P* < .05). There was no significant difference between both groups in obstructive ventilation function parameters (FEV1/FVC measured value, FEV1/FVC measured value/predicted value) (*P* > .05).

**Table 3:** Comparison of pulmonary function parameters between both groups (*x ± s*).

Index	Severe group (n = 23)	Moderate group (n = 23)	*P* value
FVC measured value (L)	2.40 ± 0.59	3.03 ± 0.65	.002*
FVC measured value/estimated value (%)	73.00 ± 13.10	81.04 ± 16.43	.079
VC_max_ measured value (L)	2.44 ± 0.60	3.03 ± 0.63	.003*
Vc_max_ measured value/estimated value (%)	73.61 ± 14.56	78.04 ± 15.86	.339
Measured value of TLC (L)	4.34 ± 0.82	5.26 ± 1.28	.007*
Measured value/estimated value of TLC (%)	75.43 ± 10.52	85.70 ± 20.44	.042*
FEV1/FVC measured value (%)	89.04 ± 6.49	83.93 ± 16.46	.183
FEV1/FVC measured value/estimated value (%)	111.13 ± 8.39	106.57 ± 12.05	.152
FEV1 measured value (L)	2.13 ± 0.50	2.59 ± 0.57	.007*
FEV1 measured value/estimated value (%)	73.70 ± 11.64	82.30 ± 15.33	.042*
MEF75 measured value (L/s)	5.03 ± 1.26	5.94 ± 1.42	.031*
MEF75 measured value/estimated value (%)	75.70 ± 13.40	80.09 ± 18.58	.373
MEF50 measured value (L/s)	3.05 ± 0.73	3.79 ± 1.49	.043*
MEF50 measured value/estimated value (%)	74.13 ± 15.28	79.35 ± 29.69	.468
MEF25 measured value (L/s)	1.47 ± 0.77	2.08 ± 0.88	.019*
MEF25 measured value/estimated value (%)	72.43 ± 29.36	93.04 ± 34.74	.039*
Measured value of DLCO (L/s)	6.23 ± 1.15	7.56 ± 2.12	.013*
Measured value/estimated value of DLCO (%)	66.57 ± 6.79	76.43 ± 21.66	.047*

FVC = forced vital capacity, TLC = total lung capacity.

* Indicates that the comparison between both groups is statistically significant (*P* < .05).

### 3.4. Correlation among GK angle, age and cardiac structure and function

The correlation analysis results of GK angle, age and cardiac structure and function parameters are shown in Table 4. GK angle in the patients with AS was significantly negatively correlated with the size of left atrioventricular cavity (LVDD, LVSD) (*P* < .05), and significantly positively correlated with diastolic function parameters (E/e’) (*P* < .05). GK angle had no correlation with other cardiac structure and function parameters (*P* > .05). Age was negatively correlated with left atrioventricular size (LAD, LVDD, and LVSD), RVD, and e’/a’ (*P* < .05), but not with other parameters (*P* > .05).

**Table 4:** Correlation among GK angle, age and cardiac structure and function.

Index	GK angle		Age	
	*r* value	*P* value	*r* value	*P* value
LAD	−0.138	.362	−0.318	.031†
LVDD	−0.304	.040*	−0.375	.010†
LVSD	−0.341	.021*	−0.299	.044†
RAD	−0.241	.107	−0.184	.220
RVD	−0.138	.359	−0.310	.036†
AO	0.097	.521	−0.080	.597
MPA	−0.143	.342	−0.105	.487
IVS	0.006	.970	−0.192	.202
LVPW	−0.101	.505	−0.128	.396
EF	−0.151	.317	0.275	.064
E/A	−0.181	.228	−0.234	.118
e’/a’	−0.206	.170	−0.295	.046†
E/e’	0.306	.039*	0.225	.133

AO = aortic diameter, EF = ejection fraction, GK = global kyphosis, IVS = inter ventricular septum thickness, LAD = left atrial diameter, LVDD = left ventricular diameter in end diameter, LVPW = left ventricular posterior wall thickness, LVSD = left ventricular diameter in end systolic, MPA = main pulmonary artery diameter, RAD = right atrial diameter, RVD = right ventricular diameter.

* Indicates that there is a significant correlation between GK angle and cardiac function indexes (*P* < .05).

† Indicates that there is a significant correlation between age and cardiac function (*P* < .05).

### 3.5. Correlation among GK angle, age and pulmonary function

GK angle of the patients with AS was significantly negatively correlated with restrictive ventilation function parameters (FVC measured value, FVC measured value/predicted value, VC_max_ measured value, VC_max_ measured value/predicted value, TLC measured value, TLC measured value/predicted value), ventilation function severity parameters (FEV1 measured value, FEV1 measured value/predicted value), and ventilation function indicators (DLCO measured value, DLCO measured value/predicted value) (*P* < .05). There was a significant positive correlation between GK angle and obstructive ventilation function parameters (FEV1/FVC measured value, FEV1/FVC measured value/predicted value) (*P* < .05). GK angle had no correlation with other parameters (*P* > .05). Age was negatively correlated with restrictive ventilation function parameters (FVC measured value, VC_max_ measured value), ventilation function severity parameters (FEV1 measured value), small airway function parameters (MEF75 measured value, MEF50 measured value, and MEF25 measured value), and ventilation function indicators (DLCO measured value) (*P* < .05). See Table 5 for details.

**Table 5:** Correlation among GK angle, age and pulmonary function.

Index	GK angle		Age	
	*r* value	*P* value	*r* value	*P* value
FVC measured value	−0.453	.002*	−0.457	.001†
FVC measured value/estimated value	−0.439	.002*	0.028	.852
VC_max_ measured value	−0.431	.003*	−0.477	.001†
VC_max_ measured value/estimated value	−0.338	.022*	0.012	.937
Measured value of TLC	−0.303	.041*	−0.250	.094
Measured/predicted value of TLC	−0.376	.010*	−0.059	.696
FEV1/FVC measured value	0.294	.047	−0.119	.431
FEV1/FVC measured value/estimated value	0.367	.012	0.059	.696
FEV1 measured value	−0.345	.019*	−0.505	<.000†
FEV1 measured value/estimated value	−0.395	.007*	−0.130	.388
MEF75 measured value	−0.258	.084	−0.319	.031†
MEF75 measured value/estimated value	−0.129	.392	−0.071	.637
MEF50 measured value	−0.201	.180	−0.360	.014†
MEF50 measured value/estimated value	−0.074	.627	−0.146	.335
MEF25 measured value	−0.175	.246	−0.319	.031†
MEF25 Measured value/predicted value	−0.271	.069	−0.183	.223
Measured value of DLCO	−0.449	.002*	−0.337	.022†
Measured value/estimated value of DLCO	−0.367	.012*	−0.055	.718

FVC = forced vital capacity, GK = global kyphosis, TLC = total lung capacity.

* Indicates that there is a significant correlation between GK angle and pulmonary function indexes (*P* < .05).

† Indicates that there is a significant correlation between age and pulmonary function indexes (*P* < .05).

## 4. Discussion

### 4.1. Influence of GK angle on pulmonary function

Most of the patients with AS and thoracolumbar kyphosis had different degrees of pulmonary function damage, and the disease mechanism was relatively complex. Among them, AS with thoracolumbar kyphosis and rib compression of the abdominal cavity reduced thoracic volume, thus damaging the ventilation function.^[8]^ In this study, the patients with AS were all associated with different degrees of thoracolumbar kyphosis, and their pulmonary functions were damaged to some extent. The larger the kyphosis angle was, the smaller the volume of the thoracic cavity and abdominal cavity would be, and the internal organs would change accordingly. Relevant studies showed that there was a certain correlation between the pulmonary function disorder of the patients with AS with kyphosis and the rotation of the diaphragm in the sagittal plane.^[9]^ According to the basic research, the patients with AS had local pathological changes in the spine and other joints, and pulmonary function decreased, which was related to NF-κB-iNOS-NO signal pathway, oxidation indicators and inflammatory factors were negatively correlated.^[10]^ In addition, the pleura was directly involved in the pathogenesis of AS, affecting the respiratory function.^[11]^ The kyphosis deformity of the patients with AS combined with ossification and fusion of costal vertebra joint, transverse costal process joint, sternoclavicular joint and spinal rigidity changes of different degrees, resulting in limited thoracic movement of patients, which could directly affect the respiratory function of patients. Berdal et al found that the lower the spinal mobility of the patients with AS was, the lower their thoracic mobility would be, and the more significant the damage of FVC% and FEV1% would be, suggesting that AS spinal stiffness could also damage the pulmonary function of patients by reducing thoracic compliance.^[12]^ In this study, the influence of GK angle on pulmonary function was evaluated when other thoracic and spinal deformities were excluded. The results showed that with the increase of kyphosis angle, the patients’ restrictive ventilation function parameters and FEV1 values decreased. The study confirmed that the larger the GK angle was, the worse the pulmonary function was in the patients with AS, and the impairment of restrictive ventilation function was significantly related to the GK angle (correlation coefficient is −0.453 to −0.303), which was consistent with previous studies.^[13,14]^ Meanwhile, this study confirmed that Cobb angle was not only related to impaired ventilation function, but also an influencing factor of small airway function and diffusion function.

### 4.2. Influence of GK angle on cardiac function

Clinical statistics show that 10% to 30% of the patients with AS have cardiovascular involvement.^[15]^ Common manifestations include conduction block, valve disease, cardiomyopathy, etc. Meanwhile, the high inflammatory state of AS leads to abnormal blood lipid metabolism and increased cardiovascular risk.^[16–19]^ Whether GK angle is an independent influencing factor of cardiac structure and function in the patients with AS has not been determined. Therefore, this study further studied the influence of GK angle on cardiac structure and function after controlling rib deformity, gender and other influencing factors. The patients with AS could often be combined with cardiovascular and other extraspinal manifestations. The patients with congenital heart disease were excluded from this study. The cardiac structural parameters of all the patients included in the criteria were within the normal range, but the atrioventricular cavity diameter of the Severe Group was smaller than that of the Moderate Group, the difference was statistically significant. Meanwhile, the correlation analysis showed that the GK angle was significantly negatively correlated with the left atrioventricular cavity diameter (LVDD and LVSD), and the correlation coefficients were −0.304 and −0.341, respectively. It confirmed that the size of the GK angle in the patients with AS could have a certain influence on the cardiac structure, which was consistent with the previous research results.^[20,21]^ The larger the GK angle was, the heavier the degree of spinal kyphosis would be, and the greater the influence on the volume of the thoracic and abdominal cavity would be. The obvious reduction of the volume of the thoracic and abdominal cavity would affect the normal activity space of the heart in the mediastinum, resulting in the smaller cardiac cavity. In this study, left ventricular systolic function parameters (EF values) of all patients were normal. Correlation analysis showed that GK angle was not significantly related to EF values, indicating that left ventricular systolic function was not affected by the degree of kyphosis. However, with the constant increase of GK angle, EF values in the Severe Group were in a downward trend compared with the Moderate Group. The study showed that the ratio of E/A and e’/a’ in the Severe Group was lower than that in the Moderate Group, and E/e’ was higher than that in the Moderate Group. The difference was statistically significant. Meanwhile, the correlation analysis showed that GK angle was significantly positively correlated with E/e’, and the correlation coefficient was 0.039, respectively. It confirmed that GK angle could have a certain influence on the left ventricular diastolic function of patients, which was consistent with previous research results.^[22,23]^ In conclusion, the GK angle in the patients with AS with kyphosis is significantly negatively correlated with the patients’ cardiac functions, mainly considering the reduction of the patients’ chest and abdominal cavity volume, reducing the range of cardiac activity, and ultimately impairing the patients’ diastolic functions.

There were several limitation of the present study, including small sample size, single center study, and 1 year study period. The future study will be performed using large sample size, multi-center study, and long study period in order to decrease the selection bias, information bias and so on.

In conclusion, after excluding the influence of thoracic malformation and gender, the GK angle of the whole spine is still the influencing factor of pulmonary function in the patients with AS kyphosis. The larger the GK angle is, the worse the patients’ restrictive ventilation functions would be, small airway function and diffusion function would be. Meanwhile, GK angle also affects the cardiac structure of the patients. The larger the GK angle is, the smaller the left atrioventricular cavity diameter will be, more greatly influencing the left ventricular diastolic function.

## Acknowledgments

This study was supported by Spinal Surgery in Beijing Dawang Road Emergency Rescue Hospital. Thank you very much for the cooperation of the patients and their families. I would like to thank the leaders of Beijing Chaoyang Emergency Rescue Hospital and the Ethics Committee for their strong supports.

## Author contributions

**Conceptualization:** Yunfei Yang.

**Data curation:** Yunfei Yang.

**Formal analysis:** Lijun Huang, Guofeng Zhao, Jiyuan Xia, Xinqi Tian, Chang Liu, Qingfu Xia.

**Investigation:** Lijun Huang, Guofeng Zhao, Jiyuan Xia, Xinqi Tian, Chang Liu.

**Methodology:** Jiyuan Xia, Xinqi Tian.

**Visualization:** Chang Liu.

**Writing – original draft:** Yunfei Yang.

**Writing – review & editing:** Yunfei Yang, Lijun Huang, Guofeng Zhao, Jiyuan Xia, Xinqi Tian, Chang Liu, Qingfu Xia.
